# A mouse model to study the C-terminal regulation of Ca_V_1.3 L-type calcium channels

**DOI:** 10.1186/2050-6511-13-S1-A50

**Published:** 2012-09-17

**Authors:** Anja Scharinger, Florian Hechenblaikner, Gabriella Bock, Mathias Gebhart, Kai Schönig, Dusan Bartsch, Anupam Sah, Nicolas Singewald, Martina J Sinnegger-Brauns, Jörg Striessnig

**Affiliations:** 1 Department of Pharmacology and Toxicology, Institute of Pharmacy, and Center for Molecular Biosciences Innsbruck, University of Innsbruck, 6020 Innsbuck, Austria; 2 Department of Molecular Biology, Central Institute of Mental Health, University of Heidelberg, 68159 Mannheim, Germany

## Background

Ca_V_1.3 voltage-gated L-type Ca^2+^ channels (LTCCs) play an important role for hearing, cardiac pacemaking and neuronal excitability. The C-terminus of Ca_V_1.3 tightly controls channel gating by an intramolecular protein interaction involving two putative α-helices (termed PCRD, DCRD), which form a C-terminal modulatory mechanism (CTM) only in full-length Ca_V_1.3 variants. In short (Ca_V_1.3_42A_ and Ca_V_1.3_43S_) Ca_V_1.3 α1 subunit splice variants CTM is absent which leads to profound changes in channel gating: activation occurs at more negative voltages and Ca^2+^-dependent inactivation (CDI) is faster.

## Methods

We quantified Ca_V_1.3 splice variants by qPCR analysis and transcript scanning, using different mouse tissues. To assess the physiological relevance of CTM, we generated a mutant mouse strain in which CTM function is disrupted by an HA-tag (Ca_V_1.3-DCRD^HA/HA^ mice). We used anti-HA antibodies to detect the expression of the HA-tagged full length channel by Western blot analysis.

## Results

The short variants Ca_V_1.3_42A_ (highest relative abundance in substantia nigra (SN) and ventral tegmental area (VTA)) and Ca_V_1.3_43S_ are both less abundant in mouse brain indicating that the full length form Ca_V_1.3_L_ comprises the most abundant form (about 50% of all transcripts). In mouse heart short transcripts are rare and Ca_V_1.3_L_ represents about 90% of all known transcripts. Ca_V_1.3-DCRD^HA/HA^ mice contain a homozygous interruption of the CTM by disrupting the DCRD helix with an HA-tag. We show that this induces "short" gating properties in this mutant full-length variant. Homozygous mice are viable and display no gross anatomical and functional abnormalities. Expression of the HA-tagged full-length channel could be detected in mouse whole brain membrane preparations. Heterozygous mice show no overt differences in locomotor activity during daytime.

## Conclusions

We have successfully generated a mouse model which will enable us to study the physiological role of CTM function *in vivo*. It mimics the (permanent) pharmacological inhibition of CTM function and will thus allow predictions about its potential as a new drug target. Furthermore, the HA-tagged α1 subunit will provide a tool to specifically determine the expression of Ca_V_1.3_L_ channels with anti-HA antibodies in mouse tissues.

